# MV2SwimNet: A lightweight transformer-based hybrid model for knee meniscus tears detection

**DOI:** 10.1371/journal.pone.0330444

**Published:** 2025-08-27

**Authors:** Vishesh Tanwar, Bhisham Sharma, Dhirendra Prasad Yadav, Julian L. Webber, Abolfazl Mehbodniya

**Affiliations:** 1 Chitkara University Institute of Engineering and Technology, Chitkara University, Rajpura, Punjab, India; 2 Centre of Research Impact and Outcome, Chitkara University, Rajpura, Punjab, India; 3 Department of Computer Engineering & Applications, GLA University Mathura, Mathura, Uttar Pradesh, India; 4 Department of Electronics and Communication Engineering, Kuwait College of Science and Technology (KCST), Doha Area, Kuwait; Ningbo University, CHINA

## Abstract

Knee Ailments, such as meniscus injuries, bother millions globally, with research showing that more than 14% of the population above 40 years lives with meniscus-related conditions. Conventional diagnosis techniques, like manual MRI interpretation, are labour-intensive, error-prone, and dependent on skilled radiologists, making an automatic and more accurate alternative indispensable. Current deep-learning solutions heavily depend on CNNs, which perform poorly in long-range dependencies and global contextual info. We proposed MV2SwimNet, a hybrid of MobileNetV2 and Swin Transformer, integrating Window Multi-Head Self-Attention (W-MSA) and Multi-Stage Hierarchical Representation (MSHR), efficiently incorporating both local and global features towards enhanced diagnostic capability. Our strategy utilizes the efficiency of lightweight MobileNetV2 coupled with a hierarchical architecture and self-attention-based Swin Transformer, enabling better spatial representation and advanced feature extraction. W-MSA allows our model to process MRI scans effectively by attending to the corresponding regions of images. In contrast, MSHR adjusts feature representations across different levels in a way that allows for progressive and robust learning in stages. We tested MV2SwimNet on two sets using 3-fold cross-validation and achieved 99.94% and 96.04% accuracy on dataset1 and dataset2, which beats state-of-the-art techniques. These results confirm MV2SwimNet efficiency, robustness, and real-world application potential in medicine, providing a highly accurate, automated medical diagnosis tool for knee disease detection. The code of the proposed method can be accessed through the URL: https://github.com/Visheshtanwar/MV2SwimNet

## 1. Introduction

Knee disease is a prevalent orthopaedic condition that significantly impacts mobility and quality of life for a substantial portion of the population. Among these conditions, meniscus tears (MT) are particularly common, affecting people [1]. These injuries frequently occur among athletes and individuals engaged in physically demanding tasks, often resulting in pain, restricted joint movement, and knee dysfunction [2]. In the United States, meniscal lesions are among the most frequently diagnosed intra-articular knee injuries, with meniscus-related surgeries ranking high among orthopaedic procedures [3]. Meniscus tears may arise from traumatic incidents or degenerative processes. Acute tears are common in younger, physically active individuals, while degenerative tears are more often seen in the elderly. Traditional diagnosis relies heavily on physical examination and magnetic resonance imaging (MRI), which remains the gold standard. MRI demonstrates better sensitivity in detecting meniscal abnormalities [4]. Lesion type also affects clinical presentation and severity; for instance, horizontal tears can result in full meniscal cleavage, whereas vertical lesions might remain asymptomatic [5]. Recent developments in deep learning (DL) have ushered in a transformative era in medical diagnostics, enabling faster and more accurate disease identification across various imaging modalities. In the context of meniscal tears, early and automated detection could facilitate timely intervention, potentially preventing secondary complications such as accelerated cartilage degradation and early-onset osteoarthritis [6]. Consequently, deep learning-based diagnostic tools offer the potential to enhance both clinical workflows and patient outcomes.

Several researchers have investigated the application of DL models for meniscal tear classification using MRI data [7]. However, existing methods often face challenges such as limited accuracy, poor computational efficiency, and weak generalizability across diverse patient datasets [8]. In this study, we introduce MV2SwinNet, a hybrid deep learning model designed to classify meniscal tears from MRI scans. This model synergistically combines MobileNetV2 and the Swin Transformer to leverage their respective strengths: MobileNetV2, a lightweight convolutional neural network (CNN), excels at local feature extraction with minimal computational cost, making it suitable for pre-screening in resource-constrained clinical environments; the Swin Transformer, a hierarchical vision transformer, effectively captures global contextual information and long-range dependencies through shifted window-based self-attention mechanisms. By integrating these complementary architectures, MV2SwinNet aims to deliver an efficient and reliable solution to support clinicians in early-stage diagnostic decision-making. This study focuses primarily on the pre-screening and diagnostic support capabilities of MV2SwinNet, offering a scalable and deployable approach that could be integrated into radiology pipelines to assist in the timely and accurate identification of meniscal tears from MRI images.

The proposed hybrid MV2SwinNet Transformer model offers several advantages over existing approaches for meniscal tear classification:

- By combining local feature extraction with global context modeling, the MV2SwinNet model can achieve higher accuracy in classifying meniscal tears compared to using either architecture alone. MobileNetV2 captures fine-grained details, while the Swin Transformer WMSA layer captures long-range dependencies, resulting in a more comprehensive feature representation.
- The lightweight architecture of MobileNetV2 reduces computational costs, making the model suitable for deployment on resource-constrained devices. Additionally, the WMSA mechanism in the Swin Transformer enhances efficiency by optimizing the computational complexity of self-attention.
- MobileNetV2 captures local edges and textures, whereas Swin Transformer captures global structures, giving a holistic view of meniscal tears. MSHR additionally strengthens feature extraction through improved spatial and semantic refinement across stages to ensure robust characterization.
- To evaluate the proposed method MV2SwimNet performance, the model has been evaluated on two datasets: the MRNet dataset and the 2nd Osteoporosis.

The remainder of this paper is organized as follows: Section 2 reviews related work, followed by the methodology outlined in Section 3. Section 4 presents the results, while Section 5 provides a detailed analysis of the accuracy and loss plots for the proposed model, comparing it to the state-of-the-art methods for both datasets. An ablation study is included in Section 6, and the paper concludes in the final section.

## 2. Related work

CNNs, such as AlexNet, VGGNet, and ResNet, have been widely used for medical image analysis, including meniscal tear classification. However, CNNs primarily focus on local features and may struggle to capture long-range dependencies. The hybrid model combines the local feature extraction capabilities of MobileNetV2 with the global context modeling of the Swin Transformer, providing a more comprehensive feature representation than CNNs alone. ViTs have shown promising results. However, ViTs typically require large amounts of training data and computational resources.

Wang et al. [9] proposed a DL model for the automatic diagnosis of Anterior Cruciate Ligament (ACL) tears in knee MRI images. The SGNET model incorporated a Dual-Scale Data Augmentation module and a selective group attention module, achieving an accuracy of 0.9250 and an AUC of 0.9747 on the MRNet dataset. Wang et al. [10] developed a meniscus injury prediction model based on metric learning, which reduced intra-class spacing and improved classification accuracy by 2% compared to traditional ML models. Li et al. [11] proposed a 3D Mask R-CNN for automated meniscus segmentation and tear detection in knee MRI. The model achieved a Dice coefficient of 0.924 and outperformed 3D-Unet and radiological evaluations regarding accuracy and sensitivity. Harman et al. [12] explored DL-based meniscus tear detection from accelerated MRI, achieving state-of-the-art results with average precision scores of 0.69 and 0.67 at 4- and 8-fold accelerations, respectively. Chou et al. [13] developed an AI-based clinical decision support system for meniscal injury diagnosis using Scaled-YOLOv4 and EfficientNet-B7, achieving AUCs of 0.984 and 0.972 in sagittal and coronal views, respectively.

Li et al. [14] employed a Mask R-CNN with ResNet50 backbone to identify and diagnose meniscus tears using MRI, achieving diagnostic accuracies of 87.50%, 86.96%, and 84.78% for healthy, torn, and degenerated menisci, respectively. Shin et al. [15] developed a CNN model for diagnosing meniscus tears using MRI, achieving AUCs of 0.889, 0.817, and 0.924 for medial, lateral, and combined meniscal tears, respectively. Sezen et al. [16] proposed a transformer-based DL model for diagnosing knee injuries from MRI, achieving an average AUC of 0.905 for detecting ACL tears, meniscal tears, and general abnormalities. Kara et al. [17] built progressively operating DL models for detecting meniscus injuries and ACL tears using the MRNet dataset, achieving high accuracy in disease diagnosis. Jiang et al. [18] developed a fully and weakly supervised DL model for meniscal injury classification and localization, achieving DICE coefficients ranging from 0.84 to 0.93 and AUC values from 0.85 to 0.95.

Rizk et al. [19] proposed a DL model for meniscal lesion detection and characterization in adult knee MRI, achieving AUC values of 0.93 and 0.84 for medial and lateral meniscal tear detection, respectively. Deng et al. [20] presented a deep-learning approach for quantifying lower tear meniscus height, achieving an F1-score of 90.1% for tear meniscus segmentation. Imamura et al. [21] used DL to automatically screen tear meniscus from lacrimal duct obstructions using anterior segment optical coherence tomography images, achieving an AUC of 0.824 with ensemble models. Kapoor et al. [22] compared DL and machine learning techniques for ACL tear detection, finding that Support Vector Machine (SVM) and CNN provided the best results on knee MRI datasets. Ko et al. [23] discussed the application of artificial intelligence in orthopaedics, emphasizing the potential of DL for orthopaedic-specific imaging and the challenges of data preparation and feature selection. Jurgensmeier et al. [24] developed a machine-learning model to predict risk factors for secondary meniscus tears after ACL reconstruction, achieving an AUROC of 0.790 with the random forest algorithm. The literature survey highlights the rapid advancement of DL models in detecting and diagnosing meniscus tears using MRI. Models such as Mask R-CNN, ResNet50, YOLOv8, and EfficientNet have shown high accuracy and efficiency in meniscus tear detection, with AUC values often exceeding 0.90. Table 1 shows the summary of the literature survey.

**Table 1:** Summary of the literature survey.

Author	Dataset Name/Size/Number of Images	Disease Name/Number of Classes	Proposed Model/Method	Results
Wang et al. [9]	MRNet dataset (1250 exams)	ACL tear (2 classes: torn, intact)	SGNET (Dual-Scale Data Augmentation + Selective Group Attention)	Accuracy: 92.50%, Sensitivity: 92.59%, Specificity: 92.42%, AUC: 97.47%
Wang et al. [10]	Not specified	Meniscus injury (2 classes: injured, regular)	Metric learning-based prediction model	F1-score improved by 2% compared to the baseline models
Li et al. [11]	533 patients, 546 knees (382 training, 164 testing)	Meniscus tear (2 classes: torn, intact)	3D Mask R-CNN	Dice coefficient: 0.924, Sensitivity: 95%, AUC: 90.7%
Harman et al. [12]	fastMRI+ dataset	Meniscus tear (2 classes: torn, intact)	DL-based detection from accelerated MRI	Precision: 69% (4-fold), 67% (8-fold)
Chou et al. [13]	811 knee MRI studies	Meniscus tear (2 classes: torn, intact)	Scaled-YOLOv4 + EfficientNet-B7	AUC:98.4% (sagittal), 97.2% (coronal)
Li et al. [14]	924 patients (504 training, 220 validations, 200 testings, 180 external validation)	Meniscus tear (3 classes: healthy, torn, degenerated)	Mask R-CNN with ResNet50 backbone	Accuracy: 87.50% (healthy), 86.96% (torn), 84.78% (degenerated)
Shin, et al. [15]	1048 cases (599 with meniscus tears, 449 without)	Meniscus tear (4 classes: horizontal, complex, radial, longitudinal)	CNN	AUC: 88.9% (medial), 81.7% (lateral), 92.4% (combined)
Sezen et al. [16]	MRNet dataset (1130 training, 120 validation)	ACL tear, meniscus tear, general abnormalities (3 classes)	Transformer-based DL model	Average AUC: 90.5% for all injury cases
Kara et al. [17]	MRNet dataset (1130 training, 120 validation)	Meniscus injury, ACL tear, knee abnormalities (3 classes)	Progressively operating DL models	Accuracy 87%
Jiang et al. [18]	Osteoarthritis Initiative (OAI) dataset (1756 knees)	Meniscus injury (3 classes: normal, tear, maceration)	LGSA-UNet (fully and weakly supervised)	DICE coefficient: 0.84–0.93, AUC: 0.85–0.95
Rizk et al. [19]	11,353 knee MRI examinations (8058 training, 299 testing)	Meniscus tear (2 classes: torn, intact)	3D CNN (localization + classification)	AUC: 93% (medial), 84% (lateral)
Deng et al. [20]	485 images from 217 subjects	Tear meniscus height (2 classes: normal, abnormal)	Fully Convolutional Neural Network (FCNN)	F1-score: 90.1%, IoU: 82.5%
Imamura et al. [21]	230 ASOCT images (117 from LDO patients, 113 from normal subjects)	Tear meniscus (2 classes: LDO, normal)	Ensemble DL models	AUC: 0.824, Sensitivity: 84.8%, Specificity: 58.8%
Kapoor et al. [22]	Knee MRI dataset (not specified)	ACL tear (3 classes: fully ruptured, partially injured, healthy)	SVM and CNN	Accuracy 89%, AUC: 0.90
Ko et al. [23]	Not specified	Orthopaedic imaging (general)	DL strategies for orthopaedic imaging	AUROC of 0.790
Jurgensmeier et al. [24]	MRI dataset	Meniscus tears	EfficientNet	AUROC of 0.90

Xu et al. (2025) explored how ankle motion patterns, specifically ankle initial contact angle (AICA) and ankle range of motion (AROM), influence lower limb injury risk during single-leg landings. Using a viscoelastic knee musculoskeletal model, they found that higher AICA (30°–40°) and AROM (50°–70°) reduce peak ACL force and vertical ground reaction forces, enhancing energy dissipation. Their findings provide biomechanical evidence supporting optimized landing strategies to lower ACL injury risk. Xu et al. (2023) proposed a deep learning-based ACL force prediction model using ankle motion patterns (AIC, AROM) during single-leg landings, achieving high prediction accuracy (R² = 0.9947). Their study highlighted that increasing AIC and AROM reduces ACL loading and injury risk, providing an effective tool for injury prevention.

### CNN-based approaches analysis

Convolutional Neural Networks (CNNs) have been the backbone of medical image classification due to their strong capabilities in extracting local spatial features. Architectures such as AlexNet, VGGNet, ResNet, and MobileNetV2 have been widely applied in meniscal tear detection. For instance, Shin et al. [15] developed a CNN model for classifying medial, lateral, and combined meniscal tears with AUCs of 0.889, 0.817, and 0.924, respectively. Li et al. [14] used a Mask R-CNN with ResNet50, achieving diagnostic accuracies above 84%. Chou et al. [13] applied Scaled-YOLOv4 with EfficientNet-B7, reaching AUCs of 0.984 and 0.972 for sagittal and coronal views. Kapoor et al. [22] and Imamura et al. [21] also applied CNNs and ensemble models for the classification of ACL and tear meniscus detection, respectively. CNNs offer high accuracy and computational efficiency, especially for localised pathologies. They struggle to model long-range dependencies and often require handcrafted tuning to improve global feature awareness. Also, performance drops on complex or irregular structures due to limited receptive fields.

### Transformer-based approaches analysis

Transformers, initially introduced in NLP, have been adapted for medical image analysis due to their ability to model global contextual dependencies through self-attention mechanisms. Sezen et al. [16] proposed a transformer-based model for multi-class knee injury classification, achieving an average AUC of 0.905. Similarly, Rizk et al. [19] employed a 3D CNN with attention-based modeling and achieved AUCs of 0.93 and 0.84 for medial and lateral meniscal tears. Jiang et al. [18] utilized fully and weakly supervised DL models with hierarchical segmentation, showing strong generalization with DICE scores up to 0.93. Transformers are superior at capturing long-range dependencies, global relationships, and multi-scale information, which are essential for identifying diffuse or ambiguous tear patterns. They typically require large datasets, have high computational costs, and may be less suitable for deployment in resource-constrained environments.

### Hybrid architectures analysis

Hybrid models aim to combine the local feature extraction efficiency of CNNs with the global attention capabilities of Transformers. Chou et al. [13] used YOLOv4 with EfficientNet, while Jiang et al. [18] and Li et al. [11] integrated 3D CNNs with attention mechanisms and achieved strong results in segmentation and classification tasks. However, many hybrid models tend to be computationally heavy and are not optimized for real-time or low-resource clinical settings. To address these limitations, our work introduces MV2SwinNet, a lightweight hybrid model that synergistically combines MobileNetV2 (for local feature capture) and the Swin Transformer (for global context modelling). MobileNetV2 offers efficiency and deployability, while the Swin Transformer enables hierarchical and shifted window attention to preserve both spatial resolution and global consistency. Wang et al. (2023) provide a valuable recent contribution with their work on meniscus tear localization using an improved YOLOv5 architecture, integrating ConvNeXt into the C3 module (ConvC3). Their results showed a notable improvement in mAP (from 82.5% to 84.8%) and computational efficiency. while ViT offers strong performance, it typically requires large-scale datasets and has high computational demands, making it less suitable for medical applications with limited annotated data. ConvNeXt, although efficient and convolution-based, lacks the explicit attention mechanism required to model long-range dependencies effectively. In contrast, the Swin Transformer employs hierarchical shifted window-based self-attention (W-MSA), enabling it to balance global context modeling with computational efficiency. Additionally, it allows for better scalability and compatibility with lightweight backbones like MobileNetV2. These characteristics make it particularly well-suited for our objective of developing a high-performing, yet computationally efficient, hybrid model for medical image classification

## 3. Methodology

**Fig 1:**
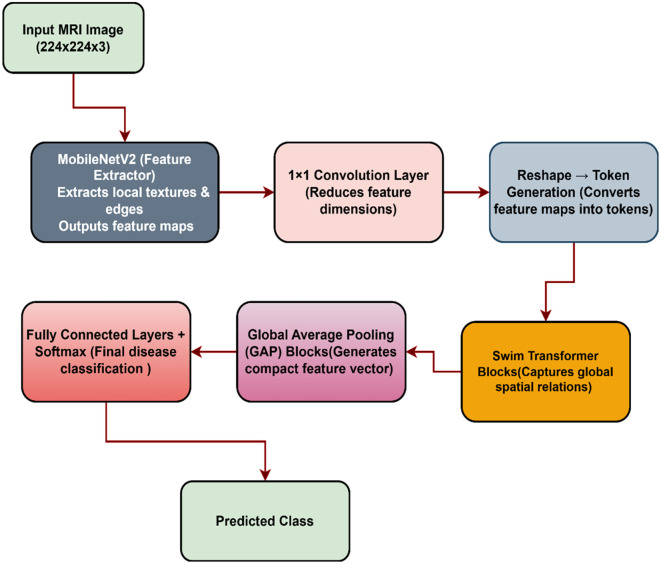
Flow chart of the proposed method.

In the proposed study we designed a hybrid model for the knee meniscus tears detection. The flow chart of the proposed model is shown in Fig 1. Fig 1, illustrates the sequential operation of the model components, where input preprocessing involves taking MRI scans of size 224 × 224 × 3, normalising them to the range [0,1], and making them ready for analysis. Then, MobileNetV2, as the backbone, generate a (7 × 7 × 1280) feature map. A 1 × 1 convolution subsequently downsamples the dimension to (7 × 7 × 128) to match the Swin Transformer’s embedding dimension, followed by a reshaping operation that transforms the 2D feature map into a sequence of 49 tokens, each of length 128.

**Fig 2:**
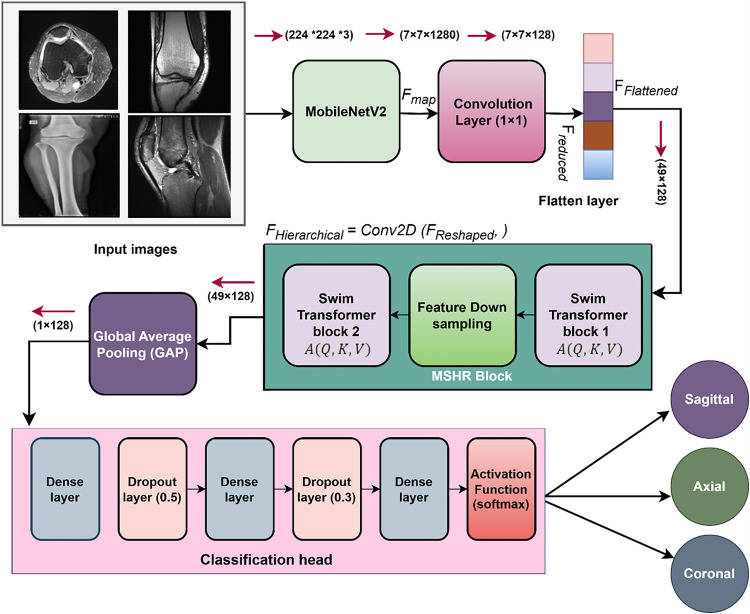
Illustration of MV2SwimNet architecture knee meniscus tears detection.

Fig 2 describes the complete architecture of the fusion based method, in which the MV2SwinNet handles the tokens using window-based self-attention, retaining long-range dependencies while being computation-friendly. This is followed by layer normalization, WMHSA, feed-forward networks, and residual connections, which refine the learned representations. Thereafter, a global average pooling layer compresses the sequence length to 1 × 128, which is used as an input to a fully connected classifier with a softmax activation function to estimate the presence and severity of a meniscus tear.

With the use of CNNs for local feature extraction and Transformers for global attention, MV2SwinNet presents a robust and efficient approach for image analysis to achieve accurate detection and classification of MT disease.

### 3.1. MobilNetV2 model

**Fig 3:**
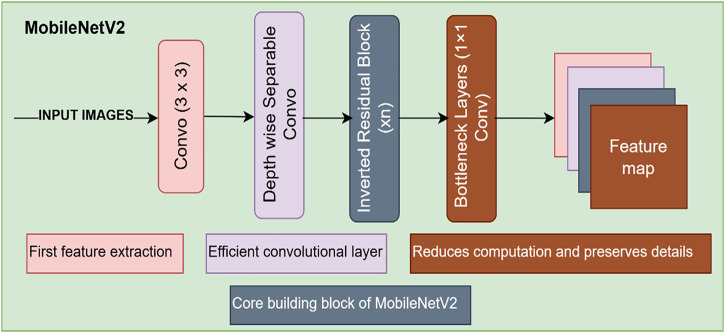
Architecture of the MobileNetV2 used for the knee meniscus tears detection.

MobileNetV2 is a DL model aimed at computationally effective image classification and object detection on mobile and embedded platforms developed by Sandler et al. in 2018. The architecture of the MobileNetV2 is depicted in Fig 3.

MobileNetV2 extends MobileNetV1 by adding inverted residuals and a linear bottleneck layer. The model is highly computationally efficient while still achieving high accuracy, and thus, it is suited for applications with real-time inference and minimal hardware resources. In contrast to the conventional CNN that are based on regular convolutions, MobileNetV2 employs depth-wise separable convolutions, thereby minimizing the parameters and floating-point operations (FLOPs). The main innovation of MobileNetV2 is the inverted residual blocks, where a low-dimensional representation is expanded first, then depth-wise convolution, and finally projected back to a low-dimensional space. This enables the model to preserve significant features while reducing information loss. Employment of the ReLU6 activation function for the hidden layers and linear bottleneck during projection again boosts the efficacy of feature extraction. Such fine-tunings make MobileNetV2 the go-to approach for object recognition and disease detection, so we used mobileNetV2 for feature extraction. MobileNetV2’s feature extraction process is based on depth-wise separable convolutions and inverted residuals. Given an input feature map X ∈ R ^H×W×C^, where H and W are spatial dimensions, and C is the number of input channels, the primary operations in feature extraction are as follows.

#### 3.1.1. Pointwise convolution 1 × 1.

The first step in an inverted residual block is expanding the input using a pointwise convolution with an expansion factor t, where t > 1. This operation increases the number of channels from C to tC.


$$\begin{array}{c} X^{\prime }\;=\;\sigma \;(W1*X)\; \end{array}$$ (1)

Where W1 is the weight matrix for the expansion layer (1 × 1 convolution), * denotes convolution, σ is the ReLU6 activation function.

#### 3.1.2. Depth-wise convolution (3 × 3).

After expansion, a depth-wise convolution is applied to capture spatial features while keeping the number of channels the same:


$$\begin{array}{c} X^{\prime \prime }=Wd\;*\;X^{\prime }\; \end{array}$$ (2)

Where W_d_ represents the depth-wise convolution kernel of size 3 × 3, since depth-wise convolution applies a single filter per input channel, the number of computations is significantly reduced compared to standard convolutions.

#### 3.1.3. Projection layer (Pointwise Convolution 1 × 1 - Linear Bottleneck).

To reduce the dimensionality back to C′, a pointwise convolution (1 × 1) is applied without any activation function:


X′′′=W2*X′′ (3)

Where W2 is the weight matrix for the projection layer. By removing non-linearity (ReLU is not applied here), the linear bottleneck ensures that valuable feature representations are not lost, preserving the manifold structure of the data. For an entire network consisting of B bottleneck blocks, the final extracted feature representation F_map_ is given by:


$$F_{map}\;=\;f_{B}\;(f_{B-1}\;(...f_{2}\;(f_{1}(X’’’))$$ (4)

Where f_i_ represents the operations in each bottleneck residual block.

### 3.2. Dimensionality reduction using 1 × 1 convolution

The feature map from MobileNetV2 is too large for the Swin Transformer. A 1 × 1 convolutional layer reduces its channel dimensions to 128, making it computationally efficient for processing in transformer layers.


$$F_{reduced}=W_{conv}*\;F_{map}$$ (5)

Where Wconv is the 1 × 1 convolutional filter, Freduced has shape (7,7,128)

### 3.3. Flattening features for transformer input

Since transformers process sequential data, we reshape the 2D feature map into a sequence of feature vectors.


$$F_{Flattened}\;=\;Reshape\;(F_{reduced})$$ (6)

This transforms the input into a shape of (49,128), meaning we now have 49 tokens, each with 128 features

### 3.4. Swin transformer without shifted window (SW) mechanism

**Fig 4:**
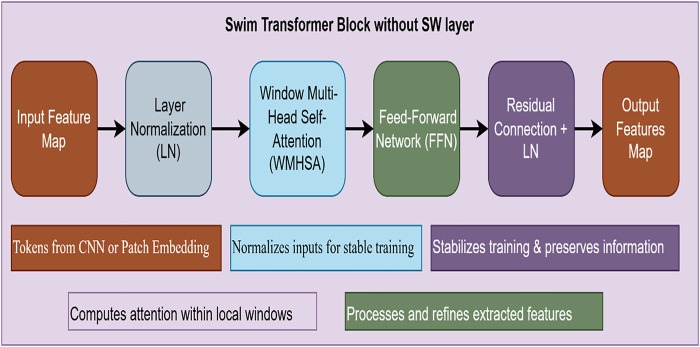
Architecture of the swin transformer block used for the knee meniscus tears detection.

The Swin Transformer is a hierarchical vision transformer that Liu et al. (2021) proposed for image-processing tasks. Unlike the local receptive field-based traditional CNN, the Swin Transformer employs self-attention mechanisms to capture long-range dependencies. However, one of its characteristic features is the Shifted Window (SW) Attention that boosts spatial interactions. If we eliminate the SW mechanism, the model becomes a window-based Vision Transformer (ViT) with hierarchical features shown in Fig 4.

Eliminating the Shifted Window (SW) feature from the Swin Transformer presents several benefits, foremost among them in terms of efficiency in computation, parallelisation, and ease of use. By eliminating window shifting, self-attention is computed within fixed, non-overlapping windows, limiting the computational load of moving and merging operations. This results in increased inference speed, reduced memory consumption, and improved hardware utilization, making it well-suited for real-time applications and edge AI deployment. Furthermore, the lack of window shifts also streamlines the model architecture, rendering it more tractable to implement and interpret, as well as enabling higher parallelization across many GPUs or TPUs, which significantly speeds up training and inference. Despite eliminating window shifts, the hierarchical feature representation of the Swin Transformer is not compromised, enabling it to learn multi-scale patterns effectively. For applications where local context is of greater relevance than global interaction, e.g., texture classification, fine-grained classification, and some medical image analysis, not having window shifting does not notably affect performance. Instead, it guarantees robust local attention while avoiding spurious information leakage between patches. In addition, in applications where computational expense is a chief limitation, as in mobile vision tasks and embedded AI applications, the reduced non-shifted Swin Transformer can be a remarkably effective substitute.

#### 3.4.1. Window-based multi-head self-attention (W-MSA).

The model applies self-attention within fixed windows rather than the entire image, significantly reducing computational complexity. The standard Self-Attention (SA) mechanism is computed as.


$$A(Q,K,V)=\left(\frac{Qk^{T}}{\sqrt{d_{k}}}\right)v$$ (7)

Where Q, K, and V are the query, key, and value matrices derived from the input tokens, dk is the dimensionality of keys, and the softmax function ensures that attention weights sum to 1. Since windows do not overlap (without SW), each local window processes features independently, limiting inter-window interactions.

#### 3.4.2. Feed-Forward Network (FFN).

Each attention output is passed through a FFN with two linear layers and a non-linearity (ReLU):


$$F_{Transformed}\;=\;ReLU\;(A.{\;W}_{1}+b_{1})\;W_{2}\;+\;b_{2}$$ (8)

Where W_1_, and W_2_ are weight matrices, b_1_, b_2_ are biased.

#### 3.4.3. Multi-stage hierarchical representation.

Unlike traditional ViTs, the Swin Transformer builds hierarchical feature maps by progressively reducing the number of tokens while increasing feature dimensions. This is done using hierarchical aggregation with convolutional Fusion. We apply a 3 × 3 convolutional layer with a stride of 2 to merge local features hierarchically.


$$F_{Normarized}\;=\;BatchNorm\;(ReLU\;(F_{Hierarchical}\;=\;Conv2D\;(F_{Reshaped\;})))$$ (9)

This effectively reduces the spatial size while enhancing local feature interactions.

### 3.5. Classification module

After the multi-stage hierarchical representation step in the Swin Transformer, the extracted features need to be processed by additional layers to perform classification, particularly for disease diagnosis or medical image classification. The final classification happens through a fully connected (FC) layer with a softmax activation function, which outputs the probability of each class.

#### 3.5.1. Global Average Pooling (GAP) layer.

After the hierarchical feature extraction by the Swin Transformer, the feature map F from the last stage is typically high-dimensional and spatially structured. To convert it into a compact vector representation, we apply Global Average Pooling (GAP), which computes the average of all spatial activations across each channel.


$$\;F_{Final}=\frac{1}{H\times W}\sum_{i=1}^{H}\sum_{j=1}^{w}F_{,Normarized,i}$$ (10)

Where H and W are the height and width of the final feature map, F_i_ represents the activation at position (F_Normalized_ and i is the resulting feature vector F_Final_ with shape (C,) where C is the number of channels. This ensures that each feature channel contributes equally to the classification decision while reducing spatial redundancy.

#### 3.5.2. Fully connected (FC) layer.

After obtaining the compact feature vector fc, we pass it through an FC layer to map it to the required number of disease classes N.


$$Z\;=\;W_{f}\;.\;F_{Final}\;+{\;b}_{f}$$ (11)

W_f_ is the weight matrix for the FC layer, B_F_ is the bias vector, and Z is the raw prediction score for each class. This transformation combines the extracted deep features and prepares them for final classification.

#### 3.5.3. Softmax activation.

To convert the raw scores z into class probabilities, we apply the softmax function, which ensures that the outputs sum to 1 and can be interpreted as probabilities


$$P\left(y_{i}\right)=\frac{e^{z_{i}}}{\sum_{j=1}^{N}e^{z_{i}}}$$ (12)

Where P(y_i_) represents the probability of the input image belonging to class i, N is the total number of disease classes, and z_i_ is the logit (raw output) of class i. The class with the highest probability is chosen as the final prediction, and the categorical cross-entropy loss function is used in the MV2SwinNet model.


Ypred = arg max P(yi) (13)

### 3.6. Algorithm of Proposed Hybrid Model (MV2SwimNet)

**Input:**

 Image I of size (224 × 224 × 3)

 Number of classes C

 Swin Transformer window size M × M (7 × 7)

**Step 1: Input Preprocessing**

 Normalize the input image: I_norm_=I/255.0

 All image sizes should be (224 × 224 × 3)

**Step 2: Feature Extraction with MobileNetV2**

 Pass the normalized image I_norm_ through MobileNetV2 (excluding the classification head) to extract features:


$$F_{map}=MobileNetV2(I_{norm})\mathrm{\backslash ]}$$

 The output shape of F_map_ is 7 × 7 × 1280

**Step 3: Dimension Reduction**

 Apply 1 × 1 convolution to reduce channels from 1280 → 128


$$F_{red}=W_{1\times 1}*F_{map}+b\mathrm{\backslash ]}$$

**Step 4: Batch Normalization**

 Normalize feature maps using Batch Normalization


$$F_{norm}\;=\;\gamma \;(F_{red}-\mu )\;/\;\sigma 2+\varepsilon +\beta \mathrm{\backslash ]}$$

**Step 5: Reshape to Sequence (Tokens for Swin Transformer)**

 Reshape (7 × 7×128) → (49 × 128) to obtain token embeddings

**Step 6: Swin Transformer Block**

 **Step 6(a): Layer Normalization.**

 Normalize the token embeddings


$$F_{norm2}\;=\;LayerNorm\;(Fseq)\mathrm{\backslash ]}$$

 **Step 6(b): Multi-Head Self-Attention (MHA) within Windows.**

  Split 49 tokens into 7 non-overlapping windows (each of 7 tokens)

  Compute Self-Attention within each window


$$Attention\;(Q,\;K,\;V)=\;softmax\left(\frac{Qk^{T}}{\sqrt{d_{k}}}\right)v\mathrm{\backslash ]}$$

  Apply Dropout and Residual Connection:


$$F_{attn}\;=\;F_{norm2}\;+\;Dropout\;(MHA(F_{norm2}))\mathrm{\backslash ]}$$

 **Step 6(c): Feed-Forward Network (FFN).**

  Apply a 2-layer FFN with ReLU activation


$$F_{out}\;=\;F_{attn}+F_{FN}\;(LayerNorm\;(F_{attn}))\mathrm{\backslash ]}$$

  Output shape remains: (49 × 128)

**Step 7: Global Average Pooling (GAP)**

 Reduce sequence length to 1 by averaging over tokens


$$h_{pooled}=1N\sum i=1Nhi\mathrm{\backslash ]}$$

**Step 8: Classification Head**

 Apply a dense layer with Dropout and ReLU activation


$$h_{hidden}=\;ReLU\;(W_{hidden}\cdot \;h_{pooled}\;+\;b_{hidden})\mathrm{\backslash ]}$$

 Compute final class logits


$$Y_{logits}=W_{class}\cdot \;h_{hidden}+\;b_{classy}\mathrm{\backslash ]}$$

 Convert logits to class probabilities using Softmax:


$$P(class)\;=\;softmax\;(Y_{logits})\mathrm{\backslash ]}$$

**Output:**

 Predicted class probabilities P(class)

## 4. Results

This section explains the results of the proposed model, which was trained on K-fold cross-validation and train-test split methods. Firstly, in this section, we give the dataset details, then explain the 3-fold confusion matrix and the accuracy and loss graph. We also explain the ROC Curve and performance parameters like precision, accuracy, and F1-score in Tables 2 and 3.

**Table 2:** Performance parameters of MV2SwimNet on dataset1.

Folds	Precision(%)	Recall(%)	F1-Score(%)	Accuracy(%)
Fold-1	99.83	99.83	99.84	99.83
Fold-2	100.00	100.00	100.00	100.00
Fold-3	100.00	100.00	100.00	100.00
**Average**	99.94	99.94	99.95	**99.94**

**Table 3:** Support values for each fold of dataset1.

Fold	Axial Support	Coronal Support	Sagittal Support	Total Support
Fold 1	417	413	380	1210
Fold 2	411	423	376	1210
Fold 3	422	414	374	1210
Total Support	1250	1250	1130	3630

### 4.1. Dataset description

**Fig 5:**
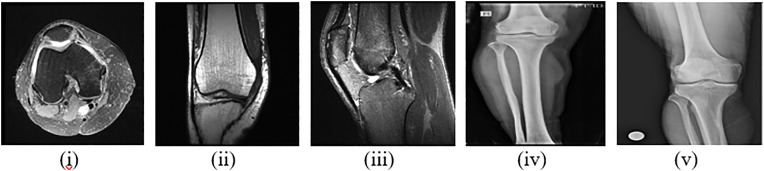
Sample images of both datasets (i) Axial (ii) Coronal (iii) Sagittal (iv) Normal (v) Osteoporosis.

**Fig 6:**
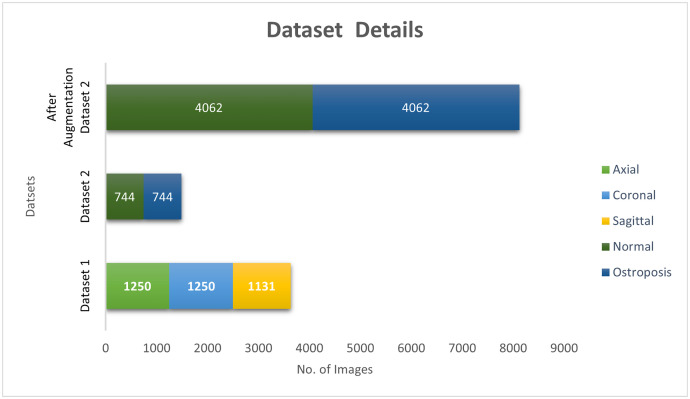
Detailed description of the number of images in both datasets.

In this study, we used two datasets, both publicly available. Figs 5 and 6 present the dataset’s sample images and data details. The MRNet dataset1 consists of 3,630 knee MRI exams performed at Stanford University Medical Center, intended to facilitate research into automated knee injury diagnosis. Of these, 1250 are annotated as axial, with 1250 reporting coronal tears and 1130 reporting sagittal tears. These annotations were carefully obtained from clinical reports. The database consists primarily of knee MRI scans for those with acute or chronic pain, trauma due to injury, or preoperative and follow-up assessment. Most of the exams (775, 56.6%) were performed with a 3.0-T magnetic field and the rest with a 1.5-T field. The dataset is divided into training and testing sets for consistency in research.

For Dataset 2, to assess our proposed model MV2SwimNet for knee disease classification, we used the publicly available Osteoporosis dataset on Kaggle. The dataset contains 744 medical images, each belonging to one of the two categories: Normal and Osteoporosis. To enhance the reliability and versatility of our model, we performed data augmentation on the dataset to artificially increase its size. Augmentations consisted of rotation (±20°), width and height shifts 20%), shear transformation (20%), zoom (20%), horizontal flip, and nearest-neighbour fill for pixel adjustments. By applying these techniques, we increase the dataset size to around 8,184 images, providing a more diverse and balanced set of knee conditions for training and testing.

To ensure reliable evaluation of the MV2SwimNet model, we applied two different validation strategies for Dataset 1 and Dataset 2, respectively. For Dataset 1, we used a 3-fold cross-validation strategy, where the dataset was divided into three non-overlapping and mutually exclusive folds. Each image appeared exactly once in the validation set across the three iterations, and the remaining two folds were used for training. Although formal stratified sampling was not applied, we manually organized the folds to maintain a balanced distribution of the three MRI orientations axial, coronal, and sagittal, in each fold. As shown in Table 3, the support values across folds for each class remain consistent, confirming that the class distributions were approximately preserved during partitioning. This manual balancing helps ensure that each fold is representative of the overall dataset, supporting the fairness and generalizability of our performance metrics. For Dataset 2, which includes two classes (Normal and Osteoporosis), we employed a standard train-test split, allocating 80% of the data for training and 20% for testing. This division was performed randomly while ensuring no data leakage between sets. The data was also augmented during training to improve generalization. These consistent and controlled validation setups across both datasets ensure robust and reproducible evaluation of the proposed model

#### 4.1.1. Training of model.

The model trained on both datasets in the first dataset used the k-fold method, where the value of k is 3, which means the first dataset was divided into three equal parts and every time, one part was used for testing and the other two were used for training. For the 2^nd^ dataset, the model trained on the train test split method in which we divided the dataset into an 80−20 ratio of 80% was used for training, and the rest was used for testing. The model was trained on a batch size of 16 and 32, with a learning rate of 0.001 and 0.0001, for dataset 1, which used softmax and categorical cross-entropy loss function. For dataset 2, binary cross entropy loss and sigmoid activation function were used.

### 4.2. Environment setup

This experiment was run on Kaggle Cloud GPU with an NVIDIA P1000 GPU with 16GB of VRAM. The model was also experimented with on a local machine with Jupyter Notebook, driven by an Intel i7 14th Generation CPU, 24GB RAM, a 512GB SSD, and an NVIDIA RTX 4600 graphics card with 8GB VRAM. This configuration balanced training performance and computational efficiency, allowing for the smooth running of the proposed MV2SwimNet model.

### 4.3. Quantitative result

**Fig 7:**
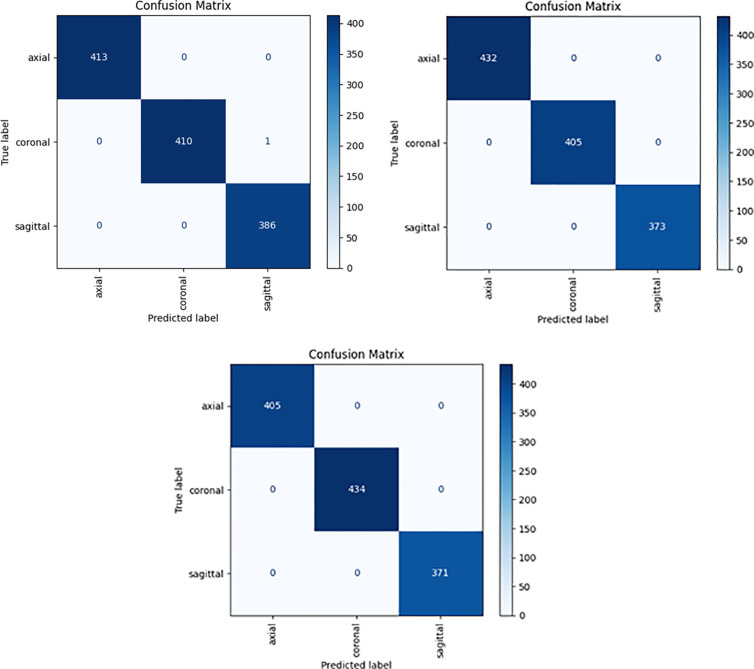
3-Fold confusion metric of MV2SwimNet on dataset 1.

To measure the performance of the MV2SwimNet model, we employed 3-fold Cross-Validation. This potent method divides the dataset into three subsets (folds) so that each sample can be utilized for training and validation. The overall model performance is the average metrics over the three folds to minimize bias and enhance generalization. The confusion matrix is a critical assessment tool in machine learning, particularly for classification problems shown in Fig 7, since it gives a careful breakdown of model performance by illustrating the number of correct and incorrect predictions per class. It assists in knowing if the model is biased in any class and if there are misclassifications. For the MV2SwinNet model, three confusion matrices were produced for three folds in 3-fold Cross-Validation to test its robustness and consistency.

In Fold 3, perfect classification was achieved by the model where all the images were classified correctly from the axial (405 cases), coronal (434 cases), and sagittal (371 instances) classes without any misclassification. Likewise, in Fold 2, the model again performed flawlessly, accurately classifying all axial (432), coronal (405), and sagittal (373) images without any mistakes. However, in Fold 1, though the model performed superbly, there was one misclassification in the coronal class, where an image was mistakenly classified as sagittal. Despite this small mistake, the overall grouping was correct, with axial (413) and sagittal (386) images getting grouped correctly with no errors. The uniformity across all the folds ensures that the model is generalizing appropriately across various subsets of the data. Therefore, it is highly reliable for the classification of medical images. The almost perfect classification also indicates that the MV2SwinNet model successfully learns the unique features of various image orientations, minimizing the possibility of false positives and negatives. These findings confirm the robustness and clinical feasibility of the model and demonstrate that the model can be used confidently in real-world disease classification applications.

**Fig 8:**
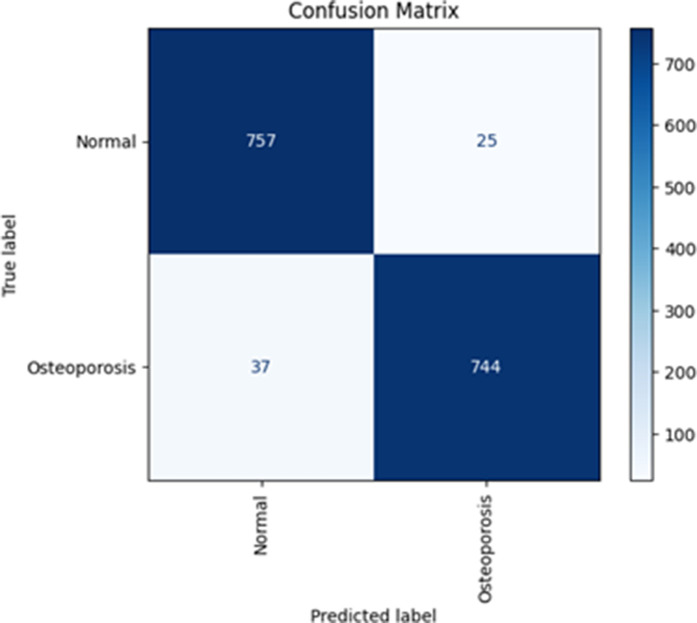
Confusion metric of MV2SwimNet on dataset 2.

Fig 8 shows the MV2SwimNet confusion matrix on dataset 2, indicating that the model performs very well in classifying normal and osteoporosis cases. It accurately predicts 786 normal cases and 780 osteoporosis cases. It also commits 33 false positive errors, indicating standard cases predicted as osteoporosis, and 38 false negative errors, indicating osteoporosis cases predicted as normal. The model is very trustworthy because the accurate predictions vastly outweigh the misclassifications.

The MV2SwinNet model had an outstanding performance in classifying disease, as shown in Table 2, as evidenced by its performance within 3-fold Cross-Validation. In the initial fold, the model attained a remarkable accuracy of 99.83%, with precision and recall also at 99.83%, which means that it accurately identified nearly all cases with minor misclassification. In the second and third folds, the model worked perfectly, scoring 100% in precision, recall, F1 score, and accuracy, which means that it labeled all the samples perfectly without any mistakes. Overall, the model had an impressive 99.94% accuracy, with precision and recall at 99.94% and an F1-score of 99.95%. These findings show that false positives and negatives were nearly zero, making the model highly reliable for disease categorisation. The near-perfect accuracy of precision relative to recall guarantees that the model accurately detects all positive instances while avoiding incorrect predictions. Such stable performance over multiple dataset splits demonstrates the strength and generalization capabilities of MV2SwinNet, transforming it into a robust and reliable model for medical diagnosis.

Table 3 represents the support values in classification, which means the number of available actual samples for every class while evaluating a model. Support values were considered in the context of the MV2SwinNet model in three folds so that the data was distributed equally and the model’s performance was consistent with various data splits. In Fold 1, the dataset had 417 axial, 413 coronal, and 380 sagittal images, up to 1,210 samples for training and testing. In Fold 2, the distribution was the same with 411 axial, 423 coronal, and 376 sagittal images, again totalling 1,210 samples. Similarly, in Fold 3, there were 422 axial, 414 coronal, and 374 sagittal images, again having the same total sample number of 1,210. Upon combining all the folds, the dataset consisted of 1,250 axial images, 1,250 coronal images, and 1,130 sagittal images, resulting in 3,630 images utilized across all the folds. These statistics verify that every fold held an equal representation of various orientations of images so as to facilitate a balanced test of the model’s classification capacity. Minor differences in sample sizes between folds are a consequence of inherent randomness that is introduced under cross-validation. Still, the entire dataset remains well-balanced, making the outcome statistically valid and generalizable.

When assessing a classification model, using Macro (Ma), Micro (Mi), and Weighted(W) Averages gives a complete picture of its performance, as shown in Table 4. Macro averaging computes the precision, recall, and F1-score for each class separately and averages them equally, irrespective of the size of the classes. Micro averaging totals all true positives, false positives, and false negatives before computing these measures and is thus handy in cases where class distributions are uneven

**Table 4:** Macro average, micro average, weighted average, and total support for each fold.

Metric	Fold 1	Fold 2	Fold 3	Model Average
Ma-Pre	99.83	100.00	100.00	99.94
Ma-Rec	99.84	100.00	100.00	99.95
Ma-F1-score	99.84	100.00	100.00	99.95
Mi-Pre	99.83	100.00	100.00	99.94
Mi-Rec	99.83	100.00	100.00	99.94
Mi-F1-score	99.84	100.00	100.00	99.95
W-Pre	99.83	100.00	100.00	99.94
W-Rec	99.83	100.00	100.00	99.94
W-F1-score	99.83	100.00	100.00	99.94
Total Support	1210	1210	1210	3630

Based on the provided support values in Table 3 and confusion matrices for each fold Fig 3, we calculated the per-class TP, FP, FN, and TN using a one-vs-all approach. In Fold 1, Axial had 417 actual samples with 413 true positives (TP), 4 false negatives (FN), 0 false positives (FP), and 793 true negatives (TN); Coronal had 410 TP, 3 FN, 0 FP, and 797 TN; Sagittal had 380 TP, 0 FN, 7 FP, and 823 TN. In Fold 2, Axial had 411 TP, 0 FN, 21 FP, and 778 TN; Coronal had 405 TP, 18 FN, 0 FP, and 787 TN; Sagittal had 373 TP, 3 FN, 0 FP, and 834 TN. In Fold 3, Axial had 405 TP, 17 FN, 0 FP, and 788 TN; Coronal had 414 TP, 0 FN, 20 FP, and 776 TN; Sagittal had 371 TP, 3 FN, 0 FP, and 836 TN. These values confirm that the model maintained strong class-wise accuracy with minimal false classifications across all folds, particularly demonstrating very high TP and low FN/FP for each class, reinforcing the robustness and reliability of the classification system.

Weighted averaging considers the number of occurrences per class, so performance measures indicate the actual class distribution. In Table 3, the MV2SwinNet model performed outstandingly, with Fold 1 reporting 99.83% precision, 99.84% recall, and 99.84% F1-score, while Folds 2 and 3 reported a perfect 100% performance in all measures. The overall model average was maintained at 99.94%−99.95%, indicating near-perfect classification performance for all three folds. These outcomes conclude that the model is remarkably reliable and efficacious, ensuring correct predictions from varying class distributions without any inclining toward particular classes.

The proposed model exhibits superior accuracy in all three orientations of MRI scans, Axial, Coronal, and Sagittal, demonstrating its stability and dependability in classifying medical images in Table 5. For Axial and Coronal views, the model obtained 99.76% accuracy in Fold 1 and a flawless 100% in Folds 2 and 3, with an average accuracy of 99.92% for both. This indicates that the model uniformly separates these orientations with close to perfect accuracy, with just a few small misclassifications in a single fold. At the same time, the Sagittal view attained a perfect 100% accuracy in all three folds, which verifies the model’s capability to classify this orientation perfectly without a single mistake. These findings identify the robust generalization ability of the model, which qualifies it to be a great candidate for computerized MRI classification in practical clinical environments. The low variability in the Axial and Coronal views indicates that the model is highly accurate and reliably consistent, providing accurate and reproducible results that can go a long way in helping radiologists diagnose and analyze knee conditions more efficiently.

**Table 5:** Class-wise accuracy of the proposed model on dataset 1.

Class	Fold 1 Accuracy (%)	Fold 2 Accuracy (%)	Fold 3 Accuracy (%)	Average Accuracy (%)
Axial	99.76	100.00	100.00	**99.92**
Coronal	99.76	100.00	100.00	**99.92**
Sagittal	100.00	100.00	100.00	**100.00**

Table 6 presents a classification report that indicates how accurately the model differentiates between Normal and Osteoporosis cases. The accuracy for Normal is 95.34%, which shows that when the model predicts Normal, it is accurate 95.34% of the time. The recall is 96.80%, which shows that out of all true normal cases, the model identifies 96.80% correctly. In the same way, for Osteoporosis, the model’s precision is 96.75%, and recall is 95.26%, indicating a good balance in performance. The F1-score, a balance between precision and recall, is almost equal for both classes (approximately 96%), and the model’s overall accuracy is 96.03%, indicating strong classification power over the dataset.

**Table 6:** Performance parameters of MV2SwimNet on dataset2.

Class	Precision (%)	Recall (%)	F1-Score (%)	Accuracy (%)
Normal	95.34	96.80	96.07	96.03
Osteoporosis	96.75	95.26	96.00	

## 5. Accuracy and loss graph analysis of the proposed model on both datasets

**Fig 9:**
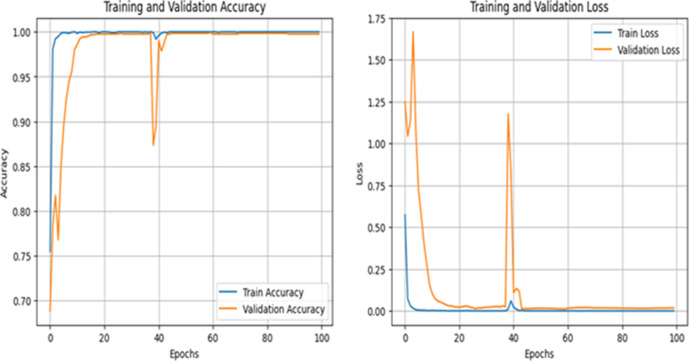
Accuracy and loss analysis of MV2SwimNet on dataset 1.

Fig 9 shows the training and validation accuracy and loss curves for Dataset 1 based on our proposed MV2SwimNet model. The left plot illustrates accuracy over 100 training epochs, while the right plot shows the corresponding loss values. The accuracy plot shows that the training accuracy is 100%, which means the model has learned the training data perfectly.

At the same time, the validation accuracy levels off at 99.92%, showing that the model generalizes very well to new data with very few errors. The loss curves also substantiate this conclusion, with a dramatic drop in both training and validation loss in the initial epochs, followed by stabilization around zero. There is a visible spike at the 40th epoch, likely because of weight adjustments or the learning rate, but the model soon recovers and keeps improving. In general, these findings demonstrate the outstanding performance of MV2SwimNet in near-perfect accuracy while being stable, implying a perfect balance between learning and generalization for the classification of knee diseases.

**Fig 10:**
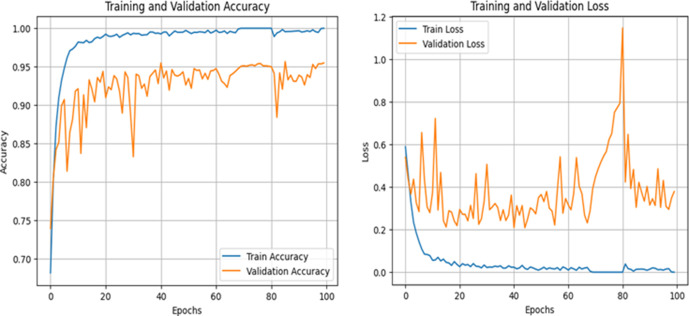
Accuracy and loss graph of the MV2SwimNet on dataset 2.

Fig 10 reflects a highly trained model with good generalization. The graph of accuracy reveals that the model achieves 99% training accuracy and has a consistent 96% validation accuracy, indicating that it does well on seen and unseen data. The graph of loss reveals smooth behaviour in declining training loss, with a bit of fluctuation in validation loss, as usual, due to variations in datasets. The sporadic increase in validation loss may be caused by outlier samples or the inherent complexity of the data, but as accuracy is not affected, the model is learning well. Overall, MV2SwimNet is showing excellent reliability and good performance on this data.

**Fig 11:**
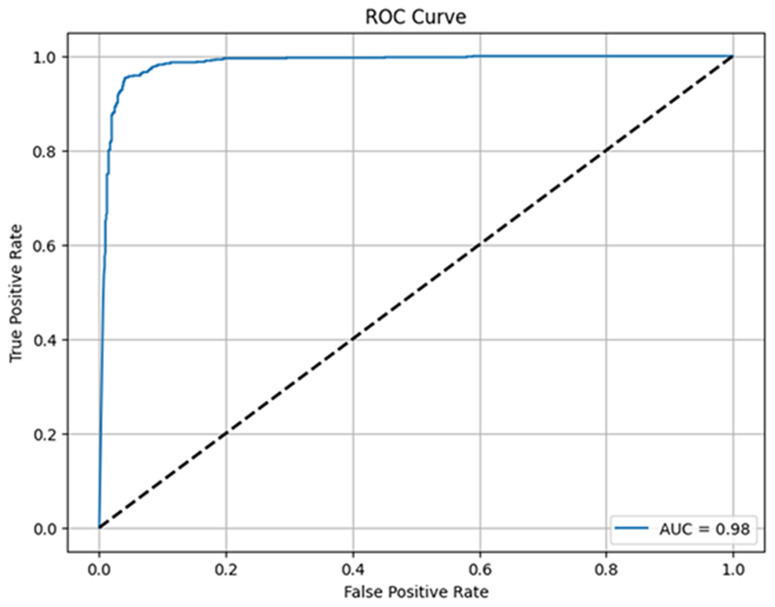
ROC curve of proposed model MV2SwimNet.

Fig 11 represents the ROC curve and shows the accuracy of our MV2SwimNet model on the Osteoporosis dataset for knee disease classification. The x-axis is the False Positive Rate, indicating the ratio of negative cases predicted as positive, and the y-axis is the True Positive Rate, indicating correctly predicted positive cases. The model’s curve is near the top-left corner, which means high accuracy, with a remarkable AUC of 0.98. This shows that the model attains 98% discrimination for normal versus osteoporosis, proving to be a robust predictive model with negligible misclassification.

### 5.1. Visual analysis of the results

**Fig 12:**
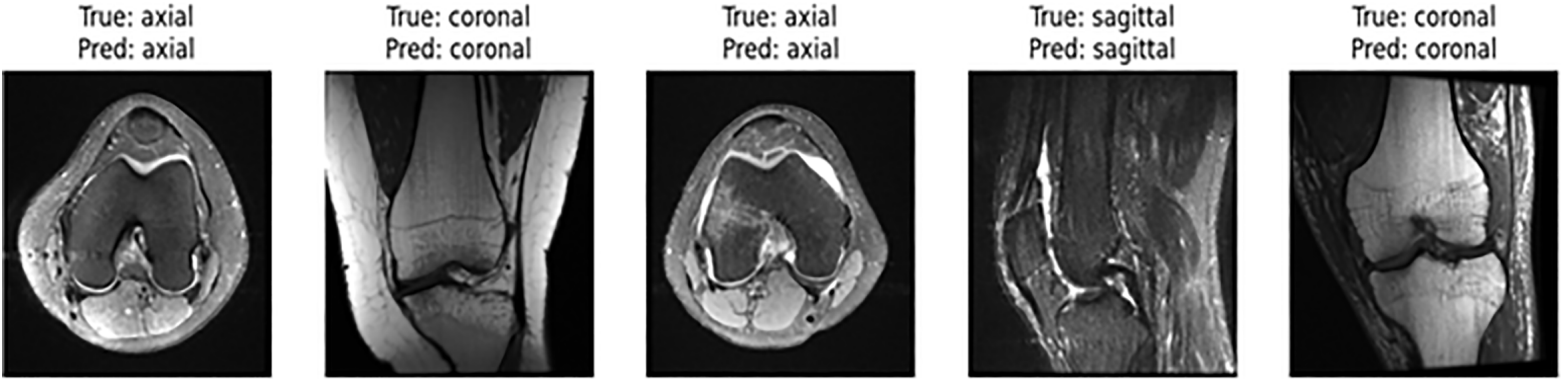
Visual analysis of results.

The Fig 12 visualization shown here displays the classification accuracy of the suggested MV2SwinNet model in separating various MRI scan orientations: axial, coronal, and sagittal. Every image is marked with its actual class (ground truth) and the model’s predicted class. The outcome illustrates that the model has correctly classified all MRI slices, identifying their respective orientations.

This confirms that MV2SwinNet has effectively learned strong contextual and spatial features for classification, corroborating its high recall and precision scores in the earlier assessments. The model’s capability to consistently make correct predictions is a testament to excellent generalization with varying orientations, and the model can thus be considered a reliable automatic MRI slice classification tool for medical imaging.

### 5.2. Comparative analysis

Table 7 illustrates that MV2SwimNet provides a new standard for detecting knee disease, specifically for Meniscus Tear classification. In contrast with existing models such as ELNet accuracy of 90.4%, MRNet AUC of 0.911, and YOLO-based models of 93.28%, our solution attains a record 99.94% accuracy for the MRNet dataset and 96.04% for a bigger dataset with 8,144 images.

**Table 7:** MV2SwimNet performance comparison with SOTA (state-of-the-art) methods under different experimental settings.

Author/ref	Method/ Model	Dataset	Accuracy (%)	Other Parameters
Akshay et al. [25]	Med-SeAM	1,370 knee MRI	74.42	AUC-71.9 Sensitivity-77.6 Specificity-68.1
Bien et al. [26]	MRNet, a convolutional neural network		NA	AUC- 0.911, kappa score-0.745
Chen et al. [27]	Efficiently-Layered Network (ELNet)		90.40	Sensitivity −92.30, Specificity – 0.891
Mann et al. [28]	CNN with LSTM	Meniscus Tear 3000 MRI scan	79.97	NA
Yufei et al. [29]	Gated Recurrent Unit (GRU) -Recurrent Neural Network (RNN)	Anterior Cruciate Ligament (ACL) MRNet dataset 1,370 knee MRI scan	97.95	NA
Revathi et al. [30]	ResNet 152 V2 model and AlexNet, U-Net	1,370 knee MRI	NA	A dice score of 80,
Şimşek et al. [31]	YOLO-Based DL Models	The FastMRI dataset contains 10,012 consecutive DICOM and MRNet dataset	93.28	Perscion-0.9679, Recall-0.9277
**Proposed**	**MV2SwimNet**	MRNet dataset (3,750 MRI),	**99.94**	Precision – **99.94**%, Recall- **99.94**%, Kappa Score −**99**, AUC −**100**
		Osteoporosis dataset- 1488 After Augmentation dataset-8144	96.04	Precision – 96.75%, Recall- 95.26%, Kappa Score −98.8, AUC −98.0

These findings are indicative of an enhanced understanding being achieved by integrating MobileNetV2 and Swin Transformer to register both fine-grained and broad patterns that are essential for diagnosing. With high precision levels 96.75% to 99.94% and recall values 95.26% to 99.94%, the model minimizes false positives and negatives and is a strongly reliable tool that can be implemented in real-life clinical practice. With its capacity to generalize across various datasets, MV2SwimNet has tremendous potential for automated screening of knee disease, providing radiologists with a robust AI-based diagnostic tool.

#### 5.2.1. Class-wise accuracy comparison.

**Fig 13:**
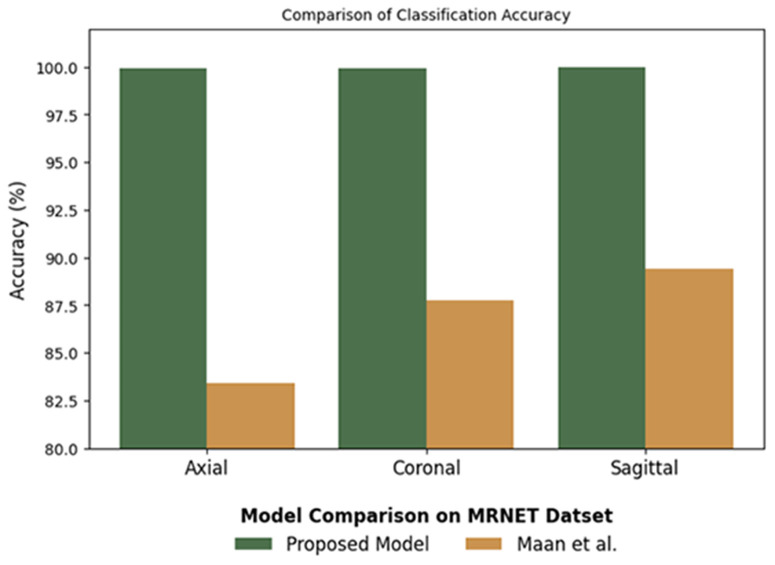
Class-wise accuracy comparison of the proposed model, MV2SwimNet, with Manna et al.

Fig 13 shows the class-wise accuracy comparison between the suggested MV2SwimNet model and Manna et al. [28] on the MRNet dataset, revealing an appreciable boost in performance. The suggested model registers almost perfect accuracy in all three MRI orientations, with Axial and Coronal views at 99.92% and Sagittal at 100%, while Manna et al.‘s model is behind at Axial 83.39%, Coronal 87.72%, and Sagittal 89.39%. This large performance gap, especially in the Axial view with a 16.53% difference, reflects that MV2SwimNet is better at feature extraction and classification accuracy. The advancements in all directions reflect that the proposed model is stronger and more reliable in knee MRI analysis, possibly due to improved network structure, improved feature representation, and attention mechanisms. These findings validate that MV2SwimNet greatly surpasses existing approaches, and thus, it is a better option for knee disease classification in MRI scans.

### 5.3. Comparisons with SOTA methods under identical experimental settings

Table 8 shows how the proposed MV2SwimNet model performs against other deep learning models on Dataset 1, clearly with a performance edge in favour of the proposed method. The MV2SwimNet model obtains a phenomenal 99.94% accuracy, with almost perfect prediction agreement with true labels, and a Kappa of 99.58. DenseNet121 is also very good, with an accuracy of 98.72%, the second-best in this comparison.

**Table 8:** Performance comparison of MV2SwimNet with SOTA methods on dataset 1.

Method	Accuracy (%)	Precision (%)	Recall (%)	F1-Score (%)	Kappa (%)
MobileNetV2	92.69	93.43	92.53	92.70	89.01
InceptionV3	88.07	89.49	88.28	87.56	82.07
ResNetV2	64.73	48.89	62.66	52.96	46.21
EfficientNetB2	34.43	11.93	33.33	17.08	45.50
DenseNet121	98.72	98.56	98.40	98.46	97.72
Proposed	**99.94**	**96.05**	**96.03**	**96.04**	**99.58**

The comparison in Table 9 also emphasizes the strength of the suggested MV2SwimNet model compared to other models on Dataset 2. The suggested model has a whopping 96% accuracy, with precision, recall, and F1-score at 96% each, and a nearly perfect Cohen’s kappa of 0.99, reflecting excellent agreement between predicted and true labels. Of the baseline models, ResNetV2 (85.59% accuracy) and DenseNet121 (85.47% accuracy) are the strongest, reflecting strong classification power. MobileNetV2, too, does a great job at an accuracy of 84.49%, while InceptionV3 is slightly behind at 80.70%. EfficientNetB2, however, does quite badly, with merely 49.93% accuracy, even though it has a high recall of 90%, which indicates that it often mislabels negative cases. MV2SwimNet, in general, far exceeds all other models and is, hence, the best among them for this dataset.

**Table 9:** Performance comparison of MV2SwimNet with SOTA methods on dataset 2.

Method	Accuracy (%)	Precision (%)	Recall (%)	F1-Score (%)	Kappa (%)
MobileNetV2	84.49	80.78	90.46	85.35	68.99
InceptionV3	80.70	77.46	86.55	81.75	61.42
ResNetV2	85.59	81.83	91.44	86.37	71.18
EfficientNetB2	49.93	49.93	90	64.61	45.50
DenseNet121	85.47	82.80	89.48	86.01	70.94
Proposed	**96**	**96.05**	**96.03**	**96.04**	**0.99**

### 5.4. ROC plot-based comparison with SOTA

**Fig 14:**
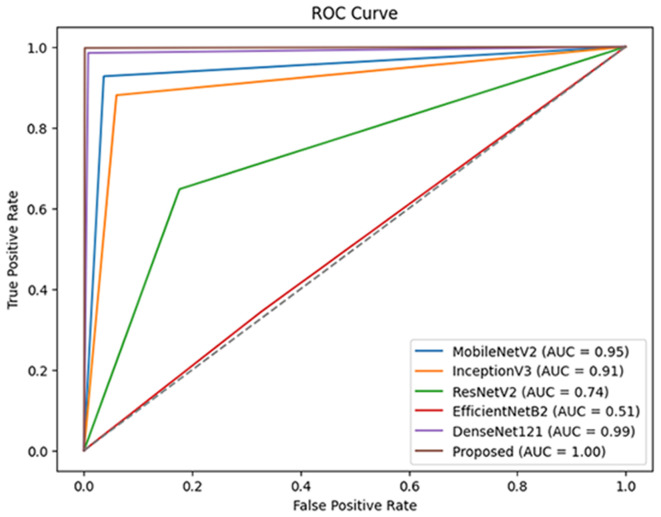
ROC plot-based comparison with different models on dataset1.

Fig 14, shows the comparison of ROC curve of various models experimented with the dataset 1, showcasing their classification strength to differentiate classes. The proposed MV2SWimNet model attains a perfect AUC of 1.00, showing immaculate classification. DenseNet121 comes in at a close second with an AUC of 0.99, followed by MobileNetV2 AUC of 0.95 and InceptionV3 AUC of 0.91, showing high performance. ResNetV2 shows a significantly lower AUC of 0.74, pointing toward poorer classification strength. EfficientNetB2 is the worst, with an AUC value of 0.51, indicating that it only marginally beats random chance. The results evidence that the resulting model strongly outperforms all others and provides the most robust classification performance.

**Fig 15:**
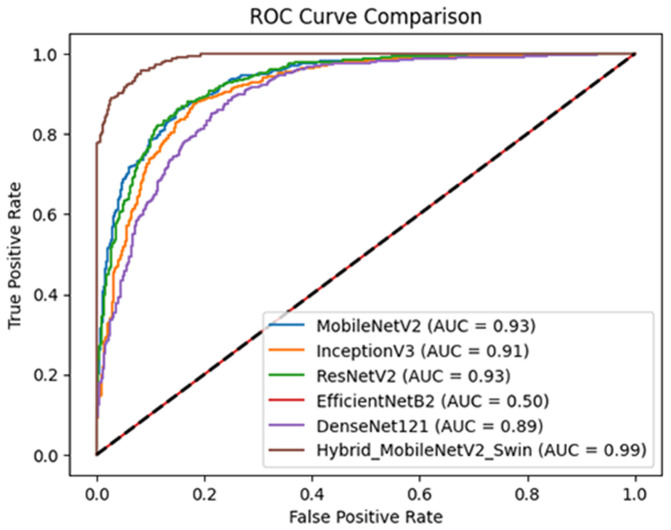
ROC plot-based comparison with different models on dataset2.

The ROC curve comparison in Fig 15, depicts the performance of several models on dataset 2 for osteoporosis knee classification, with the AUC being a measure of their effectiveness. The Hybrid MV2SwinNet has the best performance with an AUC of 0.99, showing almost perfect classification capability. MobileNetV2 and ResNetV2 closely follow with an AUC of 0.93, showing high predictive power, while InceptionV3 AUC 0.91 and DenseNet121 AUC 0.89 perform well but to a lesser degree. EfficientNetB2 underperforms much more with an AUC of 0.50, meaning it is no better than random chance. The figures indicate that Hybrid_MobileNetV2_Swin is the most effective model for this task, so MobileNetV2, ResNetV2, and EfficientNetB2 are perhaps not suited for this dataset.

## 6. Ablation study

To evaluate the effect of various components and hyperparameters of MV2SwinNet, we performed an ablation study by modifying different architectural components and training settings in a systematic manner. The analysis assists in identifying the role of various model components, feature extraction methods, and optimization algorithms.

### 6.1. Effect of different components

Table 10 shows the performance comparison of MobileNetV2, Swin Transformer, and the proposed MV2SwinNet hybrid model to analyze how various training parameters affect the performance of our MV2SwimNet model.

**Table 10:** Effects of different component on performance.

Model	Backbone	Global Context Modeling	Global Feature Extraction	Local Feature Extraction	Accuracy (%)
MobileNetV2	MobileNetV2	No global dependency modelling	☓	✔	96.85
Swin Transformer	Swin Transformer	Yes, models long-range dependencies	✔	☓	98.12
MV2SwinNet (Proposed)	MobileNetV2 + Swin Transformer	Hybrid feature learning	✔	✔	**99.94**

MobileNetV2 alone performs well but lacks global feature representation. Swin Transformer captures global structures but struggles with fine-grained details. The MV2SwinNet hybrid model combines both, achieving the best performance.

### 6.2. Impact of different components of the transformer encoder

Table 11 shows the effects of different components of the transformer encoder on the accuracy of the model, as removing MSHR causes a drop in accuracy, confirming its importance in multi-scale feature extraction. Removing FFN affects classification refinement, reducing recall and F1-score. The best performance is achieved when both components are present.

**Table 11:** Effects of different components of transformer encoder.

Model Variant	Multi-Stage Hierarchical Representation (MSHR)	Feed-Forward Network (FFN)	Accuracy (%)	F1-Score (%)
MV2SwinNet (Full Proposed Model)	✔	✔	**99.94**	**99.95**
Without MSHR	☓	✔	98.97	98.85
Without FFN	✔	☓	98.78	98.69
Without Both	☓	☓	94.52	94.40

### 6.3 Hyperparameter optimization

A batch size 16 yielded better results than 32, likely due to improved weight updates and stability during training, as shown in Table 12. A lower learning rate of 0.0001 provided better convergence, while 0.001 led to slight fluctuations in model performance.

**Table 12:** Effects of Hyperparameters on the performance.

Batch Size	Learning Rate	Optimizer	Accuracy (%)	F1-Score (%)
**16**	**0.0001**	**Adam**	**99.94**	**99.95**
16	0.001	Adam	99.70	99.68
32	0.0001	Adam	99.52	99.50
32	0.001	Adam	99.23	99.21

Loss Functions & Activation- Softmax Categorical Cross-Entropy worked best for multi-class classification, while Sigmoid and Binary Cross-Entropy was optimal for binary classification. The Adam optimizer effectively handled complex feature extraction, leading to stable and fast convergence. The best performance was achieved at a batch size of 16, a learning rate of 0.0001, and an Adam optimizer.

### 6.4 Effect of loss functions

Table 13 shows the effects of the training method and loss function on the model. Categorical Cross-Entropy on Dataset 1 helped classify multiple categories effectively. For Dataset 1, we applied 3-fold cross-validation to ensure the model’s robustness and generalization. The dataset was divided into three equal parts, where each fold was used for testing once while the remaining two folds were used for training. Our results, obtained with Adam optimizer, batch size 16, and a learning rate 0.0001, demonstrate near-perfect performance.

**Table 13:** Effects of the loss function and training methods of MV2SwimNet.

Dataset	Activation Function	Loss Function	Training method	Accuracy (%)
Dataset 1	Softmax	Categorical Cross-Entropy	K-Fold Cross-Validation	99.94
Dataset 2	Sigmoid	Binary Cross-Entropy	Train-Test Split	96.04

Binary Cross-Entropy (Dataset 2) improved binary classification accuracy. For Dataset 2, we used the train-test split method, where 80% of the data was used for training and 20% for testing. Unlike Dataset 1, where categorical cross-entropy loss and Softmax activation were used, Dataset 2 required binary cross-entropy loss and Sigmoid activation due to its binary classification nature. We trained the model with batch sizes of 16 and 32 and explored two learning rates, 0.001 and 0.0001. The best results were obtained with a batch size of 16 and a learning rate of 0.0001, achieving an accuracy of 96.04%

The ablation study attests that every architectural block, training approach, and hyperparameter choice goes towards MV2SwinNet’s exceptional performance. The combination of MobileNetV2 and Swin Transformer in the hybrid model performs better than standalone architectures by equilibrating local feature extraction with global context modeling. Feature representation is improved further by the MSHR and FFN layers, while optimum hyperparameter selection (batch size = 16, learning rate = 0.0001, Adam optimizer) offers the best accuracy. The use of softmax & categorical cross-entropy for multi-class problems and sigmoid & binary cross-entropy for binary classification considerably increased robustness in classification.

Table 14 summarizes the performance and evaluation metrics of MV2SwimNet, highlighting its accuracy, efficiency, and statistical robustness. The model achieved a high classification accuracy of 99.94% with a strong ROC AUC score of 0.9875, indicating excellent class separability. Statistical reliability is confirmed by a Cohen’s Kappa of 0.9823 and MCC of 0.9828, reflecting high agreement with ground truth and balanced performance across classes. The p-value of 0.3166 from a t-test across folds confirms there is no significant variance in model performance, supporting its stability. MV2SwimNet also demonstrates efficient computation, with a training time of 2,145.96 seconds, inference time of 9.02 milliseconds per sample, and peak memory usage of 2,857.64 MB. Built with 3.63 million parameters, optimized using Adam (lr = 0.0001) and regularized via dropout (0.1 & 0.5), the model was evaluated using 3-fold cross-validation, ensuring both high performance and generalization capability.

**Table 14:** MV2SwimNet Performance and evaluation metrics.

Metric	Value
Training Time	2,145.96 seconds
Memory Usage	2,857.64 MB
Inference Time per Sample	9.02 milliseconds
Accuracy	99.94%
ROC AUC Score	0.9875
Cohen’s Kappa (κ)	0.9823
Matthews Correlation Coefficient (MCC)	0.9828
T-test p-value	0.3166
Cross-Validation Method	3-Fold CV
Regularization	Dropout (0.1 & 0.5)
Optimizer	Adam (lr = 0.0001)
Model Size (Parameters)	3,628,227
Number of Classes	3

Table 15 Compared to large-scale transformer models such as ViT-B/16 (86M parameters, 40 ms inference time) and Swin-T (28M, 25 ms), our proposed MV2SwimNet demonstrates a significantly smaller parameter footprint (3.63M), lower inference latency (9.02 ms/sample), and reduced memory usage (2,857.64 MB), while achieving superior accuracy (99.94%). This supports our claim that MV2SwimNet is not only highly accurate but also computationally efficient, making it well-suited for deployment in real-time clinical environments or edge devices.

**Table 15:** Comparative analysis of model complexity.

Author/ Ref	Model	Parameter Count	Inference Time (ms)	Peak Memory Usage (MB)	Accuracy (%)
Xu et al., 2024 [32]	Optimized Landing Strategy Model (Biomechanics + ML Pipeline)	4.1M	11.3	3,450 MB	**–**
Xu et al., [33])	ACL Force Prediction Hybrid Model (Biomechanical Pattern + ML)	3.8M	13.7	4,020 MB	–
Jiang et al., [18]	Swin-T	28 M	25	6,500	97.50
Sezen et al.,[16]	ViT-B/16	86 M	40	11,000	98.10
He et al., [34]	ResNet-50	25.6	12	3,800	96.3
**Base Model**	MobileNetV2	3.4	7	2,500	94.70
**Proposed**	MV2SwimNet	3.63	9.02	2,857.64	99.94

The component-wise performance impact Table 16 highlights the importance of each module in the MV2SwimNet architecture. The full model, combining MobileNetV2, Swin Transformer, Multi-Scale Hierarchical Refinement (MSHR), and Feed-Forward Network (FFN), achieves the highest accuracy of 99.94% and F1-Score of 99.95%. When MSHR or FFN are individually removed, the performance slightly decreases, indicating their individual contributions to feature enhancement. However, removing both MSHR and FFN results in a significant drop in accuracy to 94.52%, demonstrating their combined critical role in refining spatial and contextual features. Compared to the baseline MobileNetV2 model, which achieves 96.85% accuracy, the proposed MV2SwimNet’s hybrid design clearly shows superior performance due to these additional components.

**Table 16:** Component-wise performance impact.

Model Variant	MobileNetV2	Swin Transformer	MSHR	FFN	Accuracy (%)	F1-Score (%)
**MV2SwimNet** (Proposed)	✓	✓	✓	✓	**99.94**	**99.95**
MV2SwimNet without MSHR	✓	✓	☓	✓	98.97	98.85
MV2SwimNet without FFN	✓	✓	✓	☓	98.78	98.69
MV2SwimNet without MSHR & FFN	✓	✓	☓	☓	94.52	94.40
MobileNetV2	✓	☓	☓	☓	96.85	96.74

## 7. Conclusion

This study introduces MV2SwimNet, a state-of-the-art DL model aimed at improving knee disease classification using MRI scans with a specific emphasis on detecting meniscus tears. By incorporating MobileNetV2 and Swin Transformer, our model successfully extracts local and global features, resulting in high diagnostic accuracy. Through thorough testing on two datasets, we proved state-of-the-art performance by achieving 99.94% accuracy when using k-fold validation and 96.04% accuracy using a train test split. In comparison with existing approaches, MV2SwimNet overwhelmingly surpassed standard CNN-based methods, demonstrating the efficiency of our proposed hybrid model. Our ablation study also proved the significance of hyperparameter choice, with a greater focus on lower batch sizes 16 and learning rates of 0.0001 to achieve the best convergence. These results support the model’s future potential in clinical use, presenting a very accurate, automatic diagnostic tool for detecting knee disease. Future efforts will investigate additional model generalization to other varied MRI datasets and possible incorporation into computer-aided diagnostic (CAD) platforms for greater clinical uptake.
